# Ultrasound in Inflammatory and Obstructive Salivary Gland Diseases: Own Experiences and a Review of the Literature

**DOI:** 10.3390/jcm10163547

**Published:** 2021-08-12

**Authors:** Michael Koch, Matti Sievert, Heinrich Iro, Konstantinos Mantsopoulos, Mirco Schapher

**Affiliations:** Department of Otorhinolaryngology and Head and Neck Surgery, University of Erlangen-Nuremberg, Waldstrasse 1, 91054 Erlangen, Germany; matti.sievert@uk-erlangen.de (M.S.); heinrich.iro@uk-erlangen.de (H.I.); Konstantinos.Mantsopoulos@uk-erlangen.de (K.M.); mirco.schapher@uk-erlangen.de (M.S.)

**Keywords:** salivary gland, salivary gland diagnosis, salivary ultrasonography, sialadenitis, salivary gland disorders, salivary calculi, inflammatory, obstructive

## Abstract

**Background**: Ultrasound is established as a diagnostic tool in salivary glands for obstructive diseases such as sialolithiasis and tumors. Concerning inflammatory diseases and in non-sialolithiasis-caused obstruction, much fewer data are available. In recent years, technical development has allowed a better assessment of the gland parenchyma, and knowledge about intraductal pathologies has increased considerably, which has provided new insights and a new interpretation of ultrasound findings. **Objectives**: To provide a comprehensive review of the literature that includes our own experiences and to point out the state of the art in ultrasound in the diagnostics of inflammatory and obstructive salivary gland diseases, taking adequate techniques and recent technical developments into consideration. **Data sources and study eligibility criteria**: A systematic literature search was performed in Pubmed using various specific key words. **Results**: According to the literature results, including our own experiences, ultrasound is of value in up to >90% of cases presenting with inflammatory and/or obstructive diseases. Technical developments (e.g., elastography) and the application of modified ultrasound techniques (e.g., transoral ultrasound) have contributed to these results. Today, ultrasound is considered a first-line diagnostic tool in these diseases. However, in some inflammatory diseases, the final diagnosis can be made only after inclusion of the anamnesis, clinical symptoms, serologic blood tests, or histopathologic investigation. **Conclusions**: Ultrasound can be considered as a first-line diagnostic tool in obstructive and inflammatory salivary gland diseases. In obstructive diseases, it may be sufficient for diagnostics in >90% of cases. In inflammatory diseases, ultrasound is at least an excellent screening method and can be used to establish the diagnosis in cases of an early suspicion. In all diseases ultrasound can contribute to better management and can be used for monitoring during follow-up.

## 1. Introduction

Ultrasound (US) as a diagnostic means was introduced into clinical medicine in the 1970s and at the beginning of the 1980s of the last century. Salivary glands were of particular interest and were addressed because of their superficial location, which can be easily approached using US.

After first reports were published, the use of US for diagnosing all kinds of salivary gland diseases aroused great interest, particularly in inflammatory and obstructive diseases. In recent publications, a clear tendency is recognizable that US is accepted as the diagnostic tool of first choice in salivary gland diseases [1,2,3,4,5,6,7,8,9,10,11,12,13,14,15,16,17,18,19,20,21,22,23,24,25,26,27,28,29,30,31,32,33,34,35,36,37,38,39,40,41,42].

Due to its non-invasive character and because it can be applied repeatedly without any irradiation, US has proven to be a useful and effective diagnostic measure, especially in children [43,44,45,46,47,48,49,50,51,52,53,54,55,56,57,58,59,60,61,62].

After remarkable developments, US today takes a leading place in the diagnostics of salivary gland diseases, as stated in relevant textbooks [63,64], and is included in several national imaging guidelines for diagnostics in obstructive sialadenitis [65,66].

While techniques like tissue harmonic imaging (THI), power mode (PM), and color-coded sonography (CCS) are part of examination routine [20,25,38,67], recent developments have pointed to new possibilities in the application of US [40], such as contrast-enhanced US (CEUS) [68,69,70,71,72], elastography [59,73,74,75,76,77,78,79,80,81], and sonohistology [82,83].

While results after the use of transfacial/transcervical US were described in the majority of reports, the value of intraoral/transoral US was investigated in only a few articles, and it was shown that additional information could also be extracted in inflammatory and obstructive salivary gland diseases [84,85,86].

In some reports, US findings were compared with those obtained after using other imaging procedures, after a combination of US with competing imaging procedures, or when these were used to supplement US to increase diagnostic information.

For the detection of sialoliths, US was shown to be of equal value and comparable to conventional radiography [87].

When sialography was compared to US, various results were described. While some publications indicated that US is inferior or at best comparable to sialography in various salivary gland diseases [88,89,90,91,92], others stated that it was of equal [93,94] or even superior value [95,96]. Regarding the evaluation of nonsialolithiasis-caused intraductal pathologies, US has proven not to be as useful when compared to sialography [87,97]. However, concerning the diagnosis of crjP [46] or Sjoegren’s disease [98], US was reported to be the most important imaging tool of first choice, and the findings obtained could be confirmed by sialography [46] or scintigraphy [98]. Because the results obtained by US were at least comparable to sialography, it was stated in some early publications that fundamental importance can be attributed to US for the diagnostics of sialadenitis [93] and that US could be at least the initial examination of choice [94].

In only a few reports was US compared to CT scan or cone-beam CT. In one, the diagnostic value of US proved to be inferior to CT scan, but the authors pointed out that both can be used complementarily to optimize further management [99]. While one report revealed that cone-beam CT had superior diagnostic value in sialolithiasis [100], in another publication, US was superior to cone-beam CT in detecting calcifications in submandibular glands [101].

In two reports, it was found that MRI-sialography compared to US was the better imaging method in detecting inflammatory and/or obstructing and salivary duct disorders [92,102].

Sialendoscopy, in comparison to US, is better suited for visualizing various intraductal pathologies as it can provide a direct view [87,92,97,100]. However, in extra- and intraductal pathologies, no matter whether they are caused by sialolithiasis or non-sialolithiasis, the simultaneous use of US and sialendoscopy can further increased diagnostic information, as described in two recent reports [103,104].

US is often performed by radiologists but may also be carried out by the treating physician. The advantages of US performed by a physician/surgeon are that all findings, namely anamnesis, clinical findings, endoscopic findings, and ultrasound findings, are handled by one person, allowing fast and effective management of the patients [105].

The aim of this review is to provide an upgrade of the development and the current state of the art of the role of US in diagnosing the relevant inflammatory and obstructive salivary gland diseases.

## 2. Methods

A systematic literature search was performed in Pubmed using the key words “ultrasound”, “sonography”, “transoral”, “transcervical”, “salivary glands”, “diagnosis”, “inflammatory disease”, “obstructive disease”, “sialolithiasis”, “non-sialolithiasis“, “color-coded sonography”, and “elastography”. All publications since the early 1970s, when ultrasound was introduced into clinical medicine, were considered. However, not all were selected. In case of duplicated publications, the publication in English was favored. Summarizing chapters in text books or review articles were considered. Publications in which no data on innovative methods or case reports were presented were not considered.

## 3. Results and Discussion

A total of 181 publications were chosen and evaluated. It is important to mention that concerning some issues, no structured data have been published up to now and are now addressed in this review.

### 3.1. Technical Aspects

When transcervical US is performed in the head and neck region, B-scan sonography, THI mode, Doppler-coded sonography, and panoramic imaging are part of the routine setting. Transducers with a frequency between 4 and 18 MHz are recommended [63,64]. Transoral or intraoral ultrasound can be a valuable supplemental technique but needs an additional transducer (e.g., 14 MHz) [84,85,86]. Enhancement of US findings can be achieved after stimulation with ascorbic acid, particularly in ductal disorders [106,107].

### 3.2. Findings in Normal Anatomy in Major Salivary Glands

The parenchyma (predominantly acinus cells organized in lobes) has a homogeneous, slightly hyperechoic sonographic tissue pattern. Connective tissues (ducts, vessels, septa) appear as more pronounced hyperechoic structures, which can be of longitudinal or punctuate shape. Due to the advanced resolution in the newer devices, tissue structures are depicted in more detail, which may result in the appearance of a “relative echo inhomogeneity” of the parenchyma and a better visualization of the normal duct system [108]. Important landmarks of the submandibular gland (SMG) are the mylohyoid muscle, the mandible, the tendon of digastric muscle, the tongue, and the tonsil region. The masseter muscle, the mandible, the posterior belly of digastric muscle, the sternocleidomastoid muscle, the retromandibular vein and the superficial temporal artery, the styloid process, and the internal jugular vein are the landmarks in the parotid gland. An accessory parotid gland is present in 20–45% according to anatomical studies [109]. The sublingual glands are of similar echogenicity and can be localized above the mylohyoid muscle in the anterior floor of the mouth [63,64].

Dost et al. [110] and Fang et al. [81] calculated the normal size of major salivary glands in European and Asian patients and published similar but not exactly equal results concerning anterior-posterior (PG 36–37 mm, SMG 34–35 mm), coronal section/depth (PG 17–23 mm, SMG 14–17 mm), and transversal section (PG 43–46 mm, SMG 23–33 mm). The shear wave velocity (SWV) in the elastography in normal glands was measured to be 1.99 m/s for the PG and 2.32 m/s for the SMG [81]. Patients presenting with enlarged glands may also have glands that appear normal. The differentiation of constitutional variations and sialadenosis from non-pathological glands can be difficult. The glands (PGs and much more than SMGs) are massively enlarged, seem to have no clearly defined borders of the gland tissue to the deeper tissue layers, and do not show any signs of obstruction [111,112]. Sialadenosis can be easily differentiated from chronic obstructive diseases by US.

### 3.3. Ultrasonographic Signs of Acute and Chronic Inflammatory and Obstructive Sialadenitis

**Figure 1:**
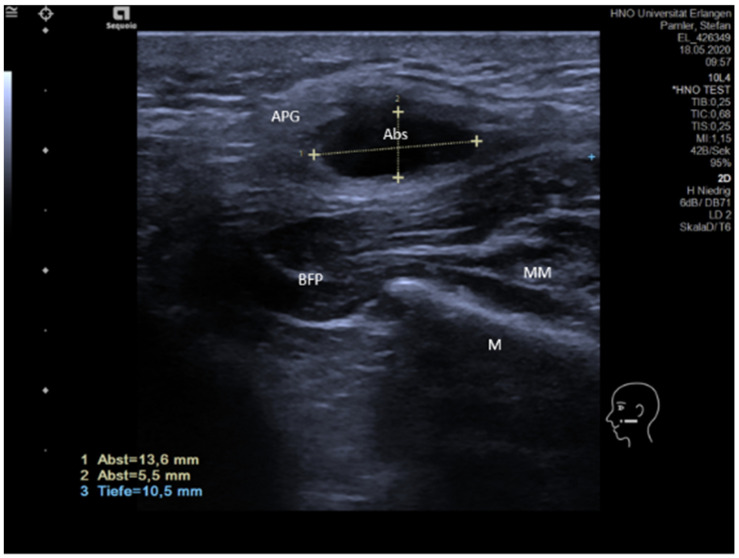
Abscess in the accessory parotid gland. A hypoechoic space-occupying lesion is visible within the gland measuring 13.6 × 5.5 mm (dotted lines). Abbreviations: APG, accessory parotid gland; M, mandible; MM, masseter muscle; BFP, buccal fat pad; ABSC, abscess.

Acute or subacute inflammation and obstruction are both characterized by more or less pronounced hypoechoic changes of the gland tissue [63,64]. While inflammation is primarily caused by increased perfusion and secondarily by ductal obstruction, the situation is opposite in duct obstruction. The hypoechoic changes can be differentiated by applying color-coded US. An abscess can develop as a complication and is characterized by a hypoechoic or echo-free space-occupying lesion with distal enhancement of the shadow (Figure 1). Hyperechoic internal reflexes can be present as signs of air-producing microbes. A sialocele may have a similar aspect in US but is characterized by different clinical symptoms. Ultrasound-guided puncture may be helpful to differentiate these.

**Figure 2:**
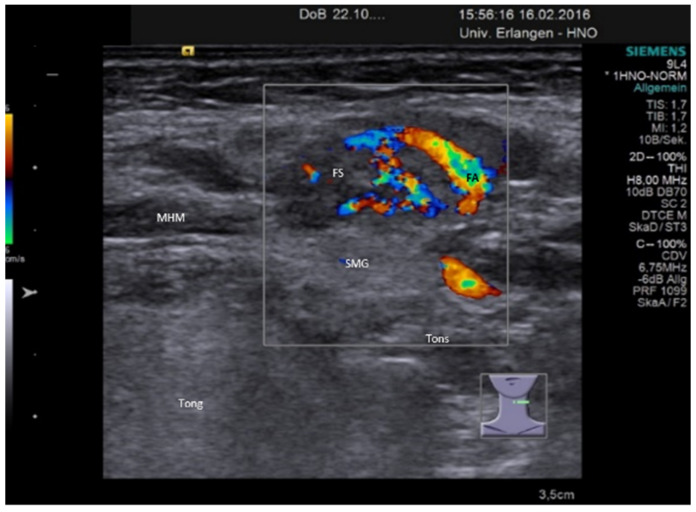
Focal fibrosis in a left SMG. The parenchyma is partially hypoechoic changed. In contrast to any tumor-like or tumorous lesions, the tissue structure is not changed. The perfusion pattern is regular and not different to the pathological and or the residual uninvolved gland. Abbreviations: SMG, submandibular gland; MHM, mylohyoid muscle; Tong, tongue; Tons, tonsil; WD, Wharton’s duct; FA, facial artery; FS, focal sialadenitis.

Chronic sialadenitis is characterized by a tendency towards a smaller gland size [113]. The parenchyma tends to be diffusely hypoechoic but appears more often as heterogeneous with a mixed echo structure. The coarse aspect corresponds to diffuse cirrhotic parenchymal changes. Duct dilation may be present optionally depending on the cause (e.g., obstruction by a stenosis). Perfusion mostly appears somewhat increased or due to the rarefication of gland tissue but may also be even decreased. The changes can involve the whole gland or parts of the gland depicting a focal hypoechoic area with no significant disturbance of the architecture of the gland parenchyma (Figure 2). By the less pronounced hypoechoic tissue texture and the unchanged gland tissue architecture, these focal changes can be differentiated from focal infiltrations caused by autoimmune or granulomatous diseases (see below). The glands may appear small and hard on palpation, and if SMGs are affected, the term “Kuettner’s tumor” is used encompassing various unspecific [113] or specific causes.

#### 3.3.1. Acute or Subacute Primary Non-Obstructive Microbial Sialadenitis

**Figure 3:**
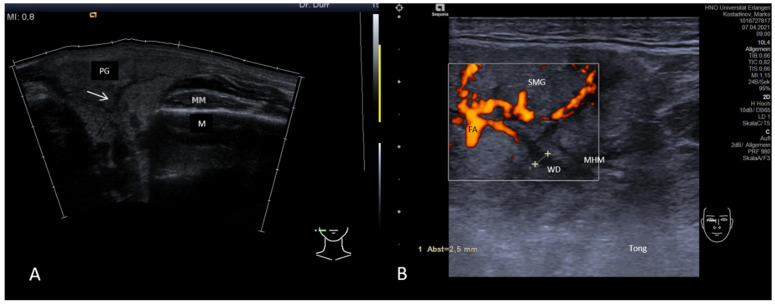
Acute viral sialadenitis in the PG (**A**) and in the SMG (**B**). The volume of the gland shows a tendency towards enlargement. The parenchyma is hypoechoic mainly by a spongy aspect (white arrow), which is caused by duct dilation and increased perfusion, which cannot be distinguished by plain US. Doppler-coded sonography was used to differentiate between duct dilation (Wharton’s duct dilation measuring 2.5 mm, dotted line) and vascular structures (low-flow perfusion indicated by Power Doppler, **B**). Abbreviations: PG, parotid gland; SMG, submandibular gland; M, mandible; MM, masseter muscle; MHM, mylohyoid muscle; Tong, tongue; WD, Wharton’s duct; FA, facial artery.

Subacute or acute inflammation caused by infectious agents such as viruses or bacteria may lead to acute gland swelling. Patients usually present with unilateral swelling, possibly also with putrid secretion. The parenchyma often shows a hypoechoic tissue texture with a spongy aspect, mainly caused by edema and increased vascularity (Figure 3A,B). Ducts are not affected primarily, but possibly secondarily, and may show dilation. Gland vascularity is mostly markedly increased. Differentiation between duct dilation and increased perfusion can be made by application of CCS. A spongy aspect, in particular in viral infection, is caused more by increased perfusion than dilated ducts (Figure 3A,B). However, in viral infection, gland echotexture may also appear normal, with multiple enlarged intraparenchymal lymph nodes as the most relevant pathologic sign [114].

Acute bacterial purulent sialadenitis may show increased vascularity and duct dilation, and any cause of duct obstruction, in particular sialolithiasis, has to be excluded in such cases.

#### 3.3.2. Obstructive Sialadenitis Caused by Sialolithiasis

Sialolithiasis makes up 60–85% of all cases presenting with obstructive sialadenitis. As well the parenchyma (sialadenitis), the duct system is also involved (sialodochitis, duct dilation). Bilateral glands are involved in about 10% of cases. In US, the glands are enlarged with a hypoechoic parenchyma. Direct signs are bright, hyperechoic reflexes mostly with clear distal shadowing, which may be less pronounced in some cases. Duct dilation is often present as an accompanying indirect sign. Today, US is considered as the imaging tool of first choice in sialolithiasis in adults and children [9,47,54,103,115,116,117,118,119,120,121,122,123,124,125,126,127,128,129,130]. Depending on their consistency, stones with a size of 1–2 mm can be diagnosed but may also be overlooked up to a size of 3–4 mm [122,126]. Sensitivity was reported to be 70–95%, with better results in recent publications [9,36,65,89,100,102,131,132]. The observed PPV was 94–96% [63,64,100,126,131,132].

**Figure 4:**
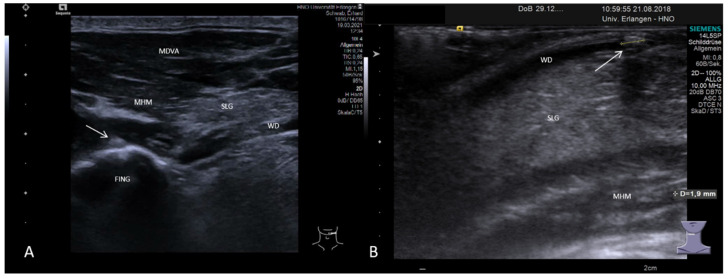
Two modifications of US application to overcome the extinction of the sound waves by the mandible in order to diagnose small sialoliths in the distal Wharton’s duct. A small stone is depicted using transcervical US and “sonopalpation” (**A**). The small stone (white arrow) is moved out of the shadow of the mandible with the palpating finger. Application of transoral US is shown in (**B**). A small stone (white arrow, size 1.9 mm, dotted line) near the papilla is shown after positioning of a small transducer onto the mucosa of the floor of the mouth. Abbreviations: SLG, sublingual gland; MHM, mylohyoid muscle; DMVA, digastric muscle venter anterior; WD, Wharton´s duct; FING, palpating finger.

In a recent publication, we investigated the value of US in diagnosing sialolithiasis after investigation of more than 2250 glands. Sensitivity, specificity, accuracy, PP value, and NP value were all in the range of 93–96%. It was remarkable that a duct dilation could be observed in 95% of all false-negative findings, which can serve as an indirect sign of sialolithiasis [132]. If not present primarily, duct dilation can be provoked by stimulation of the gland secretion, e.g., after administration of ascorbic acid. Stones may then be visible only after such an enhancement [106,107]. As our results indicate, the great majority of false-negative results were observed in small, sometimes less mineralized stones, which were located in the distal duct in both major salivary glands or within the hilum of the PG [132]. If transcervical US is used for submandibular stones with distal location, the transducer should be positioned longitudinally and be rotated medially around the lateral body of the mandible to enable a straight and not a tangential view of the region of interest to avoid acoustic shadowing of the mandible. Besides this simple clinical inspection and simultaneous palpation, examination by transcervical US, referred to as “sono-palpation” [133] (Figure 4A), or application of transoral ultrasound [84,85,86] (Figure 4B) were described. If the diagnosis or differential diagnosis continues to be unclear, consecutive [132] or even simultaneous [103] application of sialendoscopy was valuable for establishing the correct diagnosis in difficult cases.

Due to its less invasive or even non-invasive character, examination with US, if used in its various applications and modifications, can detect sialolithiasis in nearly all cases. One frequently mentioned concern regarding routine use of ultrasound is that it is dependent on the experience of the examiner. An investigation of interrater reliability performed by our study group did not confirm this. The evaluation of findings, which were stored as video clips, were anonymized and sent to several different examiners for evaluation. Cohen’s Kappa and Fleiss Kappa coefficients were 0.76 each, and a pairwise concordance of 88% between examiners was observed. The results indicated that US, in particular when video documentation is performed, is associated with an acceptable interrater reliability when compared to other imaging methods [130,134].

Due to the higher invasiveness and/or accompanying irradiation, competing imaging tools such as sialendoscopy [131,132,135] or CT-scan/cone-beam CT-scan [99,100] do not seem to be the first choice, although their sensitivity for the diagnosis of sialolithiasis was increased compared to US in several studies.

Differential diagnosis (DD) includes intraductal calcifications (e.g., scarring tissue, foreign bodies), extraductal calcifications (e.g., vascular malformations, calcifying lymph nodes), or pneumoparotid [130,131,132,136].

In cases in which DD is obviously difficult, ultrasound-guided diagnostic techniques [41,103] and the simultaneous application of US and sialendoscopy added diagnostic value, the latter by providing a singular, real-time, three-dimensional intra- and extraductal overview [103]. Besides this, US provides diagnostic information in the preparation [103,137], as support in the treatment [103,138,139,140,141,142,143,144,145] and in the (post-therapeutic) follow-up [69,79,146] in sialolithiasis.

**Figure 5:**
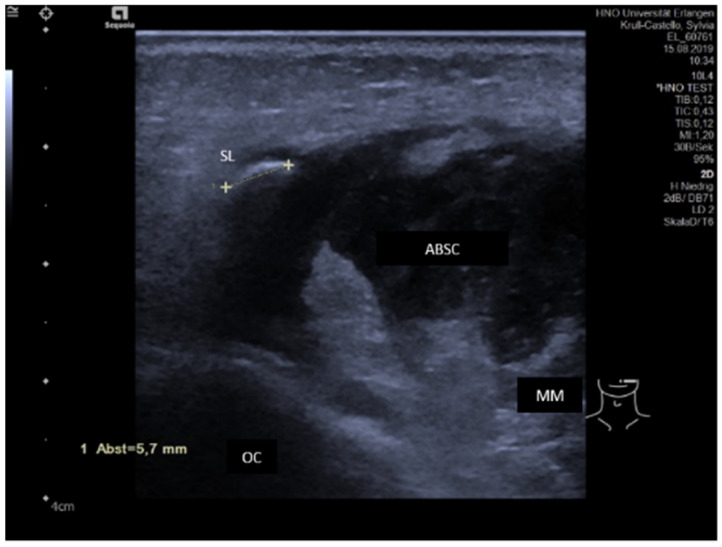
Abscess formation of the left cheek region. The transcervical view to the cheek shows a hypoechoic to anechoic space-occupying lesion overlying the middle and distal part of Stensen’s duct. The formation was irregularly shaped with ill-defined borders and small intra-lesional hyperechoic reflexes representing thick secretion and/or air-producing bacteria. Sialolithiasis is recognized as the main reason and must always be excluded. A sialolith was also visible in this case characterized by a hyperechoic reflex measuring 5.7 mm (dotted line). Abbreviations: MM, masseter muscle; OC, oral cavity, SL, sialolith; ABSC, abscess.

Sialolithiasis may lead to acute purulent sialadenitis, which may develop to an intra- or periglandular abscess and/or abscessing lymphadenitis. The gland is enlarged and has a hypoechoic parenchyma which includes a hypoechoic fluctuating mass, possibly including internal bright echoes caused by air-producing aerobic bacteria (Figure 5). Sialolithiasis is the most frequent cause of abscess formation and must be excluded in any abscess formation.

#### 3.3.3. Non-Sialolithiasis-Caused Unspecific Sialadenitis with Sialodochitis and/or Duct Stenosis with Primary or Secondary Obstruction

Non-sialolithiasis-caused unspecific inflammatory salivary disease usually presents as sialadenitis accompanied by duct inflammation (sialodochitis) and/or duct stenosis. A total of 80% of cases with sialodochitis and 75% with duct stenosis occur in the parotid gland. No disease that can be associated with sialodochitis or stenosis is known, and sialolithiasis is not present.

Symptoms can be caused by duct inflammation (sialodochitis) or duct stenosis. Sialodochitis can be secondarily obstructive due to fibrinous plaque formation. Except by our study group [108,147], sonographic criteria for craS/craP with sialodochitis and/or stenosis have not been investigated intensively and systematically until now, and no criteria were elaborated that can be used to distinguish between them.

In a recent publication, we assessed ultrasound findings in cases presenting with sialodochitis, with duct stenosis, and without duct anomaly and in duct stenosis with duct anomalies. Ultrasound findings could be better interpreted after inclusion of the knowledge obtained by sialendoscopy [108]. If sialodochitis was present, the parenchyma was hypoechoic in nearly 80% of the cases, and duct diameters were significantly higher (mean 0.7 mm, range 0–4.3 mm) compared to glands which did not show any duct inflammation (0.3 mm, range 0–2.7 mm). No significant differences were observed when the mean duct diameters of stenoses not associated with duct anomalies and sialodochitis cases were compared. The obstructive plaques causing duct dilation are difficult to identify by ultrasound and may have a similar effect in bends, encroachments, and duct diversions as in duct stenosis. If stenoses associated with duct anomalies were included, the diameters were significantly higher (mean 4.1 mm, range 0–19 mm, see below) compared to sialodochitis or stenosis without duct anomalies [108].

**Figure 6:**
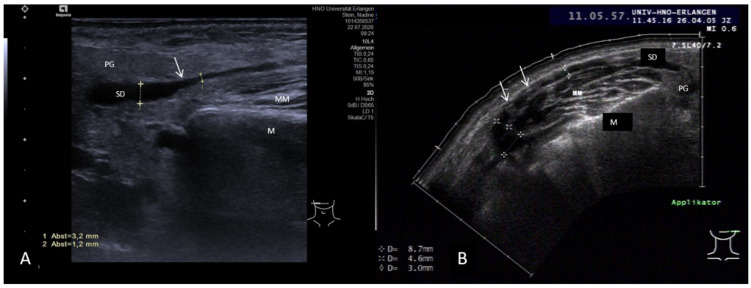
(**A**,**B**) Appearance of sialodochitis in two cases. A clear and sudden change (white arrow) in the lumen of Stensen’s duct with an increase from 1.2 to 3.2 mm is recognizable in (**A**). The parenchyma of the PG is slightly hypoechoic. (**B**) shows a case of chronic parotitis accompanied by duct dilation with an irregular shape of the duct wall caused by circular and semicircular encroachments. Due to their consistency (connective tissue), such encroachments appear as slightly hyperechoic structures (white arrows). These correspond to webs and/or strictures observable in sialendoscopy. The diameter of the duct can be very variable during its anatomical course. The parenchyma shows a tendency to be hypoechoic. Abbreviations: PG, parotid gland; M, mandible; MM, masseter muscle; SD, Stensen’s duct.

Sialodochitis may appear in US as a pronounced duct dilation and a tendency to form a megaduct due to a sudden change in the caliber of the ductal lumen (Figure 6A) or may be associated in other cases of craP with encroachments of the duct without a stenosis effect. The duct then has somewhat irregular changes in the duct wall along its course (Figure 6B).

**Figure 7:**
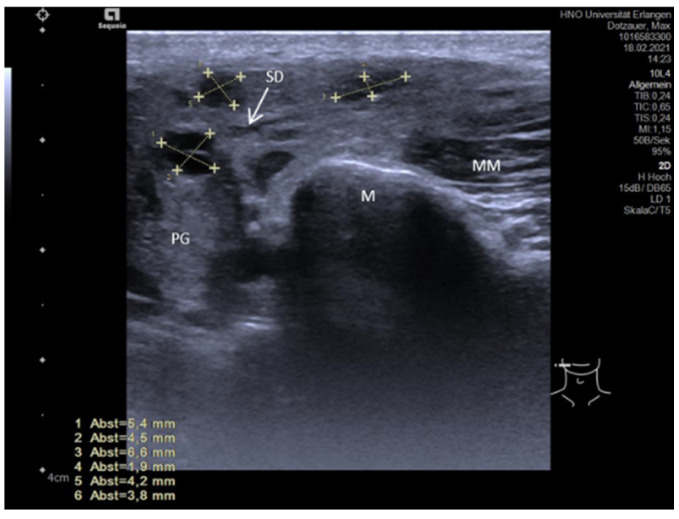
A case of chronic recurrent parotitis with sialectatic sialodochitis is shown. The parenchyma is slightly hypoechoic. No pronounced dilation of the main parotid duct is recognizable (white arrow). Multiple nearly anechoic formations are visible. Because no internal echoes were detectable, but connections to the main duct system were noted, the presence of sialectatic sialodochitis was diagnosed. Abbreviations: PG, parotid gland; M, mandible; MM, masseter muscle; SD, Stensen´s duct.

In chronic adult sialectatic parotitis, another aspect of sialodochitis is visible. Multiple hypoechoic, spot-like formations are recognizable, which provide a polycystic aspect caused by the duct-ectasias. Within the sialectasia, hyperechoic reflexes, often without a clear extinction of sound waves, can possibly be visualized, indicating air located within the sialectasia (Figure 7). If significant distal shadowing is visible, small calcifications or calculi, which are difficult to distinguish, may be present. HIV infection has to be considered as DD, as it may also be characterized by polycystic tissue changes appearing as “black spots”, but shows an otherwise unremarkable aspect of the gland parenchyma [148,149].

**Figure 8:**
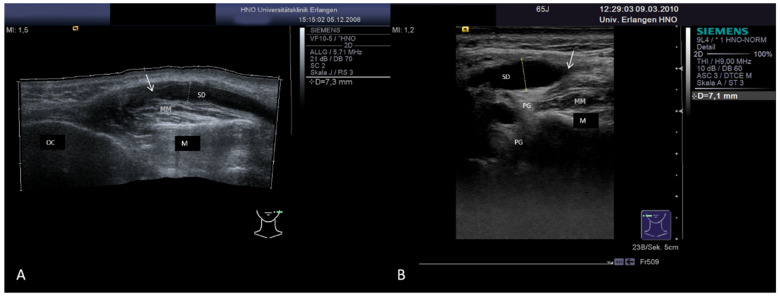
(**A**,**B**) Two examples of ductal stenosis within the Stensen’s duct system. (**A**) indicates a massive duct dilation measuring 7.3 mm (dotted lines) at the transition of the distal to the middle part in a left PG. The duct lumen could not be clearly identified more distally to this region (white arrow). Figure (**B**) shows a massive change in the caliber of the duct at the transition of the middle to the proximal duct system. While the proximal diameter can be measured (7.1 mm, dotted line), a ductal lumen is much more difficult to identify distally. In both cases, the location of the stenosis can be estimated, distally in (**A**) and proximally in (**B**).

Duct stenoses make up 5% of all obstructive diseases in the SMG and up to 25% in the PG. They are mostly located unilaterally, but in 5–15% of cases can occur bilaterally, in particular in the PG. Patients report more or less painful gland swelling. Glands are typically enlarged in the US examination. However, in longstanding obstruction with recurrent episodes of sialadenitis, the gland volume may be decreased as a sign of insufficient gland function, which correlates with markedly reduced secretion. As duct stenosis can be found in 75–80% of all cases in the PG, it was more intensively investigated. A characteristic finding in stenosis is a hypo- to anechoic longitudinal, cylindrical, or oval formation along the course of the duct with distal enhancement, but without significant extinction of the shadow. Duct dilation may be enhanced after stimulation of the gland secretion [106,107]. Slight hyperechoic tissue texture within the stenotic area without clear shadowing may indicate connective or scarring tissue. The larger the diameter, the higher the probability of the presence of a duct stenosis. Although the differentiation of stenosis not associated with duct anomalies from sialodochitis proved to be difficult, the presence of a duct stenosis is very likely if the duct diameter is >4–5 mm. A megaduct is present if the duct diameter is ≥5 mm, and in these cases, a stenosis can be suspected with high probability (Figure 8A,B) [108,147]. While the grade of the luminal narrowing cannot be estimated even by measuring the extent of dilatation, the location of a stenosis within the duct system can be roughly calculated (Figure 8A,B).

**Figure 9:**
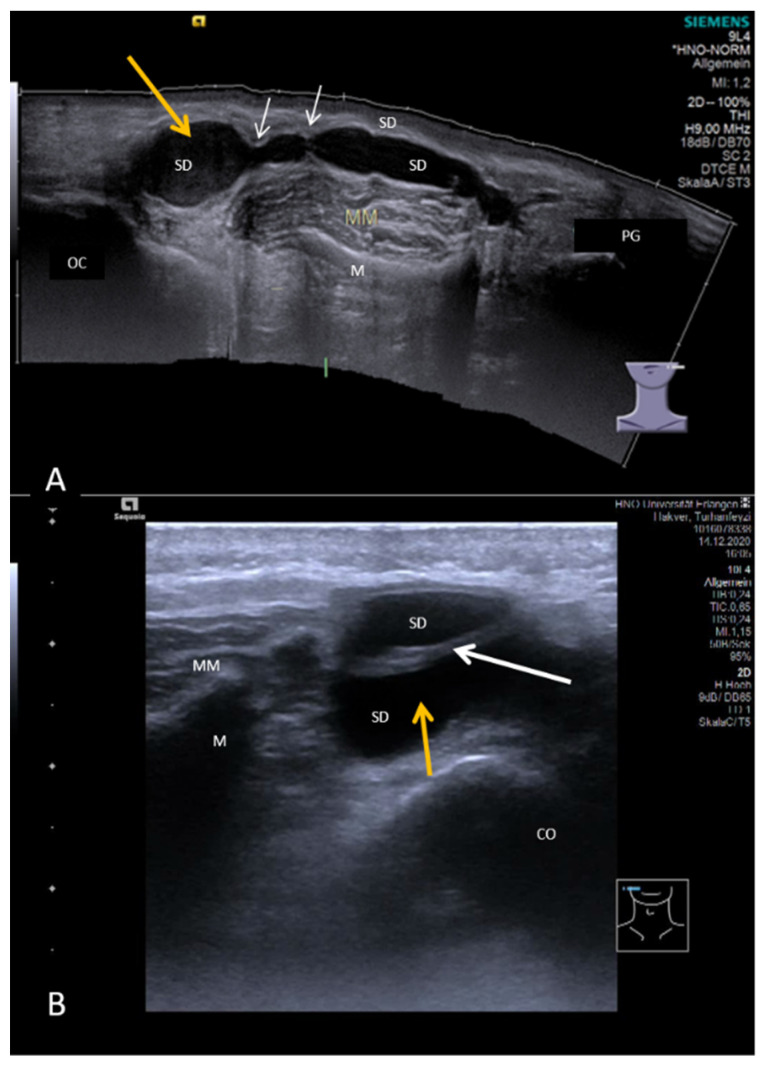
(**A**,**B**) Duct stenosis with ductal variations in a left (**A**) and a right (**B**) PG. The stenosis is nearly always located distally. The distal duct diameter is often massively increased to more than 10 mm (yellow arrows) in the context of a megaduct. The stenotic effect is caused by a more or less pronounced duct stenosis in the distal duct in combination with ductal abnormalities like pronounced-to-extreme duct kinks observable in sialendoscopy (connective tissue, white arrow, **B**) or circular and semicircular encroachments (connective tissue, white arrows (**A**)). The diameter of the duct can be very variable during its anatomical course. A tendency to form a megaduct may be observable up to the intraparenchymal duct system, which may have a polycystic aspect here. The parenchyma of the PG is mostly slightly hyperechoic and does not appear changed in a pathological way. Abbreviations: PG, parotid gland; M, mandible; MM, masseter muscle; SD, Stensen’s duct; OC, oral cavity.

As shown by a recent publication of our study group, parotid duct stenosis with or without an association with distinct duct anomalies can be differentiated by US concerning the echotexture of the parenchyma, the extent of duct dilation, and morphological criteria based on anatomical changes. Besides this clear morphologic US, criteria were elaborated concerning stenosis with ductal anomalies and by transoral US more precise details of distal stenosis/kinking could be visualized (Table 1, Figure 9A,B) [108,147]. Abbreviations: PG, parotid gland; M, mandible; MM, masseter muscle; SD, Stensen’s duct; OC, oral cavity.

The diagnosis of a duct stenosis without duct anomaly, which seems to have a stronger inflammatory component, is much more difficult than the diagnosis of stenosis with duct anomalies, which seem to have less inflammatory with clearly visible anatomic changes of the duct system. The parenchyma is significantly more often hypoechoic in stenosis without duct anomalies. Duct diameters in stenosis with duct anomalies were significant higher compared to sialodochitis and duct stenosis without duct anomalies. When ROC curves were calculated, a cut off value of 2.1 mm was associated with a sensitivity of 74.2% and a specificity of 90.2% for stenoses without duct anomalies, and a cut off value of 5.1 mm was associated with a sensitivity of 92.6% and a specificity of 96.8% for stenoses with duct anomalies [147]. Considering this, the differentiation of stenoses with duct anomalies from sialodochitis and stenoses without duct anomalies was easy to perform due to significant differences [108].

Intraductal application of contrast-enhanced ultrasound (IA-CEUS) has been described as another valuable alternative in visualizing obstructive diseases, in particular non-sialolithiasis-related duct obstruction, but detailed information concerning possible underlying pathologies has not been provided [68].

It has to be mentioned that some relevant findings could be better interpreted after deriving knowledge obtained from sialendoscopy, e.g., the meaning of the hyperechoic formations though the course of the duct (semicircular/circular encroachments, Figure 6B) or within the distal duct system, which were identified as massive duct kinks contributing to stenotic effects (Table 1, Figure 9B). As in sialolithiasis [103], the simultaneous application of US and sialendoscopy increased the diagnostic value by providing a singular, real-time, three-dimensional intra- and extraductal overview in cases with duct stenosis and can also be helpful in their management [104,150]. DD comprises unspecific or specific intra- or periglandular lymphadenopathy.

Radiotherapy or radioiodine therapy causes a specific kind of obstructive sialadenitis. In the early stages, it often looks like sialodochitis, and duct stenosis develops in the later course. The tissue texture of the parenchyma can have a wide spectrum ranging from a nearly normal appearance to a pronounced hypoechoic and heterogeneous aspect. Duct dilation mostly does not exceed 2–3 mm, as it is caused mainly by fibrosis of the duct wall in combination with decreased gland function due to irradiation effects. The typical gland tissue and acinus cell lobes are replaced step by step by heterogeneous tissue, which may end in fatty and fibrous degeneration indicating gland atrophy (see below).

### 3.4. Disease-Associated Inflammatory Sialadenitis with Sialodochitis (and Possible Secondary Obstruction)

As in non-disease-related sialadenitis, US may show variable aspects. Tissue texture of the parenchyma can appear nearly normal or can be hypoechoic to a slight to pronounced extent. Moderate duct dilation (2–4 mm) may be visible and the DD of stenosis may be difficult, in particular in less pronounced stenosis. The differentiation from non-disease-associated sialadenitis or chronic sialadenitis without sialectasia can be difficult or even impossible. However, in such cases, US can give an indication of further evaluation and management for establishing a specific diagnosis, in particular in the case of a suspicious anamnesis.

#### 3.4.1. Chronic fibrinous Sialodochitis

**Figure 10:**
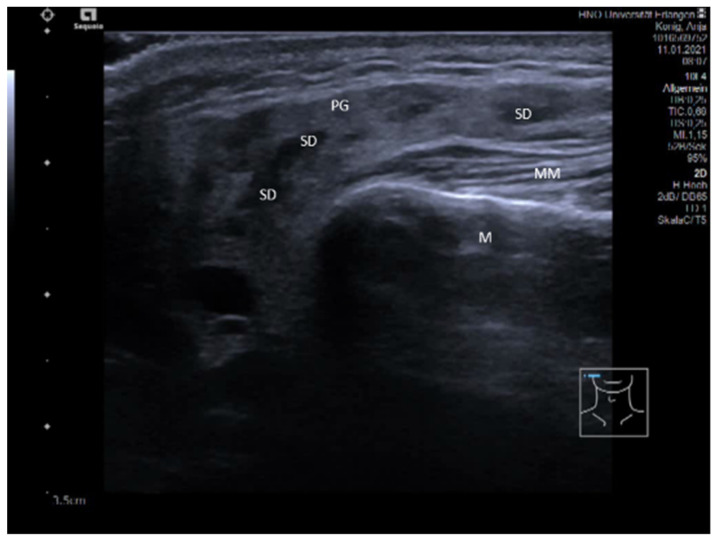
Eosinophilic sialodochitis in the right PG. The parenchyma is hypoechoic and characterized by multiple anechoic formations within the parenchyma corresponding to multiple dilations of Stensen’s duct, giving it a “leopard-skin” appearance. These are oriented along the course of the duct and do not typically have a rounded shape as in sialectatic sialadenitis and are not perfused. Abbreviations: PG, parotid gland; M, mandible; MM, masseter muscle; SD, Stensen’s duct.

In chronic recurrent parotitis with fibrinous or eosinophilic sialodochitis (aka Kußmaul’s disease), obstructing fibrinous plaques with infiltration of eosinophils and massive duct dilation with a tendency to form a megaduct can be observed. In many cases bilateral glands or even all major salivary glands are involved (Figure 10). US findings are less valuable for primary diagnosis or DD, but can be used for further monitoring. No reports dealt with the role of US in eosinophilic sialodochitis, and findings were obtained mainly by conventional sialography or MR-sialography [151]. In connection with the history of allergic disease and the presence of hallmarks in US, fibrinous sialodochitis can be suspected and further diagnostic measures were initiated (e.g., gland biopsy).

#### 3.4.2. Chronic Recurrent Juvenile Parotitis

**Figure 11:**
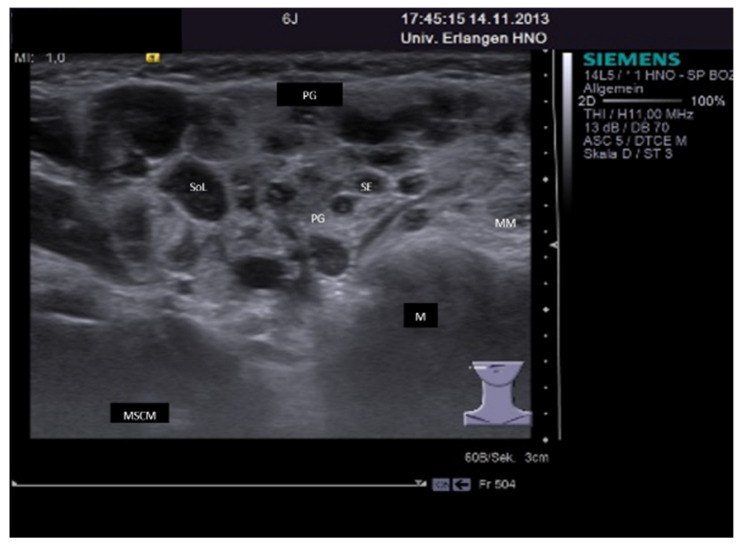
CrjP in a right PG (6-year-old child). The gland is enlarged, and the gland parenchyma is slightly hypoechoic with multiple pronounced hypo-echoic areas, providing a “leopard-skin”-like aspect. Some of the “spots” are nearly echo-free with small hyperechoic reflexes in some parts of the duct corresponding to sialectatic parts. Others are more solid space-occupying lesions corresponding to an infiltration of lymphocytes and plasma cells. Abbreviations: PG, parotid gland; M, mandible; MM, masseter muscle; MSCM, sternocleidomastoid muscle; SoL, space occupying lesion; SE, sialectasia.

Chronic recurrent juvenile parotitis (crjP) is diagnosed in childhood (predominantly at the age of 1–3 years) and at a juvenile age. Recurrent episodes of unilateral or bilateral painful swelling are reported. Considering this, a fast and reliable diagnosis can be made by US, which is the method of choice [46,48,49,50,55,57,152]. Sonographically, the glands are enlarged with mixed echogenicity. The parenchyma is heterogeneous and appears like a “leopard-skin” (Figure 11). Hypoechoic areas (mainly due to infiltration of lymphocytes) and multiple hypoechoic or nearly anechoic spots with a polycystic-like aspect are visible (mainly due to peripheral duct ectasia).

Hyperechoic reflexes with “dirty shadowing” can be observed within the sialectatic ducts, possibly corresponding to air. Sialoliths have not been observed in crjP. US findings in connection with the young age of the patients strongly suggest the diagnosis of crjP. HIV infection is an important DD, as a similar polycystic aspect is visible. In contrast to crjP, the gland is otherwise unremarkable with a normal appearance of the parenchyma [148,149]. Unspecific or specific intraglandular lymphadenopathy may accompany crjP. Juvenile Sjoegren’s disease is rarely observed. Both represent possible DDs.

#### 3.4.3. Sjoegren’s Syndrome/Disease

Sjoegren’s syndrome (SS) can be found unilaterally or bilaterally or even in all glands. It is diagnosed more in PGs than in SMGs. Gland secretion is clear, sometimes slightly murky. At later stages, it may be reduced due to gland function insufficiency. Depending on the stage of the disease, glands can be enlarged or be of normal or reduced size. US was addressed as the most relevant diagnostic tool in SS, but also in general in sialadenitis caused by or related to the autoimmune-system, in textbooks [63,64] and multiple publications after examination of adults [67,78,153,154,155,156,157,158,159,160,161] and children [56,61,62,162,163,164]. In general the parenchyma has a mixed cystic or solid echo-texture with a “leopard-skin”-like or cloudy aspect.

According to the literature and our experiences, the ultrasound criteria of SS seem be stage-dependent [63].

**Figure 12:**
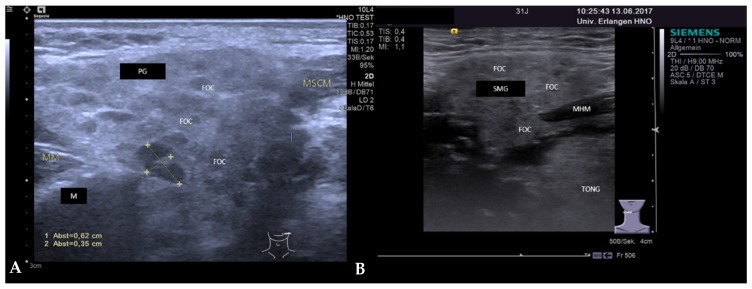
(**A**,**B**): Ultrasound findings at the early stage of M. Sjögren in the PG (**A**) and SMG (**B**). The parenchyma is slightly hypoechoic with multiple small (size 2–3 mm) and more pronounced hypo-echoic foci, which correspond to an accumulation of lymphatic cells. Glands are moderately enlarged. Abbreviations: PG, parotid gland; SMG, submandibular gland; M, mandible; MM, masseter muscle; MHM, mylohyoid muscle; MSCM, sternocleidomastoid muscle; TONG, tongue; FOC, Focus.

Salivary gland US was reported to be a highly specific tool for the early diagnosis of primary SS. Compared to controls, patients with primary SS showed a significantly higher salivary gland US score, which indicates the changes in the echostructure of salivary glands [165]. Our experiences confirm this. At the early stage, diffuse, only small-punctate hypoechoic changes can be observed, which may be difficult to recognize but may be the only indication of the presence of the disease because even serology may not be conclusive at this time (Figure 12A,B).

In one meta-analysis, including 14 publications, the pooled sensitivity and specificity was reported to be 75–85% and 88–95% for various salivary gland ultrasound scoring systems. Small hypoechoic punctate changes (as depicted in Figure 12A,B) were counted. The authors concluded that the 0–4 scoring system had a higher specificity and a lower heterogeneity compared to all other systems described and suggested that it may be used as a universal salivary gland ultrasound diagnostic standard [166].

At the later stages, more pronounced hypoechoic to anechoic parenchymal changes appear solid due to infiltration of lymphocytes or cystic due to a beginning loss of functional tissue resulting in sialectasia. In some cases, multiple hyperechoic reflexes are present, corresponding to micro-calcifications within the parenchyma or ductectasia. Sialoliths were observed in rare cases.

**Figure 13:**
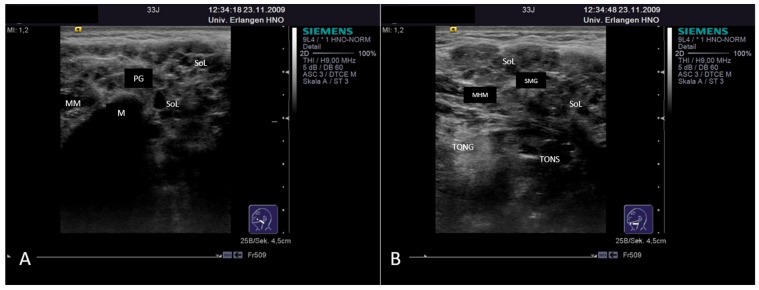
(**A**,**B**): Ultrasound findings at the advanced stage of M. Sjögren in the left GP (**A**) and left SMG (**B**). The parenchyma is markedly heterogeneous and replaced by hypoechoic to anechoic spot-like formations as less parenchyma is recognizable. Hypoechoic areas appear more solid and may correspond to an infiltration of lymphatic cells, anechoic to sialectatic ducts after atrophy of the acinic cells. The glands are enlarged. Abbreviations: PG, parotid gland; SMG, submandibular gland; M, mandible; MM, masseter muscle; MHM, mylohyoid muscle; TONG, tongue; TONS, tonsil; SoL, space-occupying lesion.

At the advanced and late stage, glands may appear smaller, and the parenchyma is often rarified and dominated by hypoechoic solid or cystic changes. Secretory function is then markedly reduced (Figure 13A,B).

**Figure 14:**
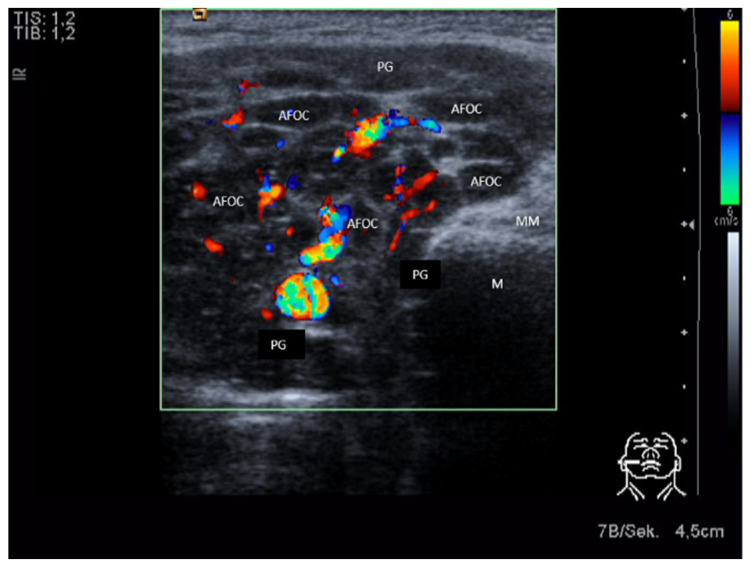
Acute stage of M. Sjögren in a right-sided PG. The gland is massively enlarged. Hypoechoic areas (active foci) are visible throughout the parenchyma and represent a massive infiltration of lymphatic cells The echotexture appears honeycomb-like. Perfusion is massively increased. Regular gland tissue is nearly not visible any more. Abbreviations: PG, parotid gland; M, mandible; MM, masseter muscle; AFOC, active focus.

As the disease progresses, patients may experience flare ups of disease activity. If there is an inflammatory flare up, the glands are typically massively enlarged and the parenchyma shows a pronounced hypoechoic to anechoic texture with a “honey-comb-like” texture. These changes are caused predominantly by a massive infiltration of lymphocytes and are accompanied by a massive increase in perfusion (Figure 14).

**Figure 15:**
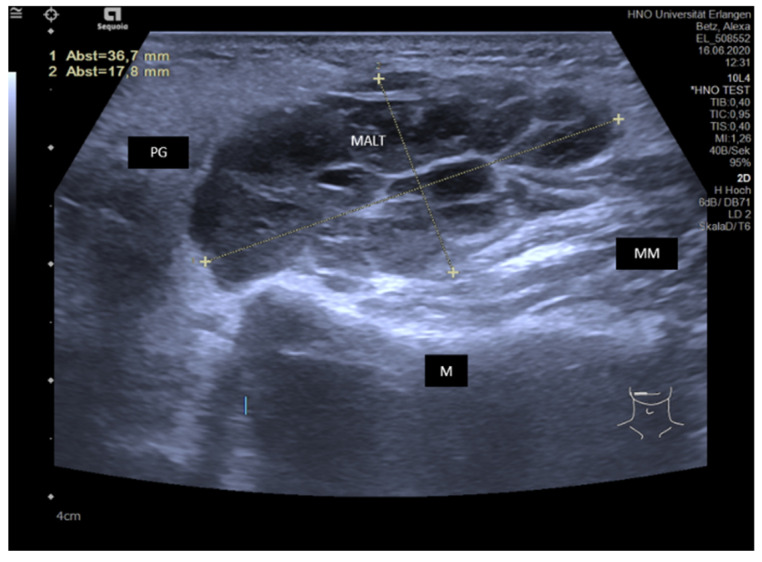
Histologically proven MALT lymphoma in a PG on the right side. A hypoechoic and lobulated, polycyclic space-occupying lesion with hyperechoic septal internal reflexes is depicted, measuring 36.7 × 17.8 mm. The lesion showed an increase in size during repeated follow-up investigations. Abbreviations: PG, parotid gland; M, mandible; MM, masseter muscle; MALT, MALT lymphoma.

As MALT lymphoma occurs in 5–6% in the course of the disease, intraglandular lymph nodes must be monitored regularly at 6-monthly intervals. Massive enlargement of intra- or periglandular lymph nodes with a hypoechoic to anechoic tissue pattern and a significant change in the anatomical architecture may point to the presence of MALT lymphoma (Figure 15).

There is a consensus that, because US criteria have been described as good, US findings are of enormous value for the diagnosis of SS, and US is accepted as first-line screening tool for suspected SS [56,61,154,155,160,166]. It has also been useful for identifying secondary juvenile SS in patients presenting with mixed connective disease [164]. US is also regarded as an adequate method for monitoring and surveillance concerning the development of MALT lymphoma [63,64]. In one study, elastography was applied in patients with SS. Increased SWVs were measured, but this seemed to indicate chronic glandular inflammation rather than fibrotic changes, and therefore the diagnostic value was not increased [78].

Some authors have suggested that US may replace other diagnostic techniques [155]. Others compared the results of salivary gland US using various accepted scoring systems with results obtained after labial biopsy. It was shown that the sensitivity, specificity, accuracy, and AUC-ROC values after salivary gland US were comparable with the results after gland biopsy. However, the final diagnosis currently relies on clinical data, evaluation by scoring systems, serology and, if necessary, by gland biopsy. It must be awaited whether US scores find their way into a classification for the diagnosis of SS.

Due to the heterogeneous changes in the parenchyma, intra- or periglandular lymphadenopathy, cjrP, and HIV with its polycystic changes in an otherwise unremarkable gland [148,149] belong to the relevant DDs.

#### 3.4.4. IgG4-Associated Involvement of Salivary Glands

**Figure 16:**
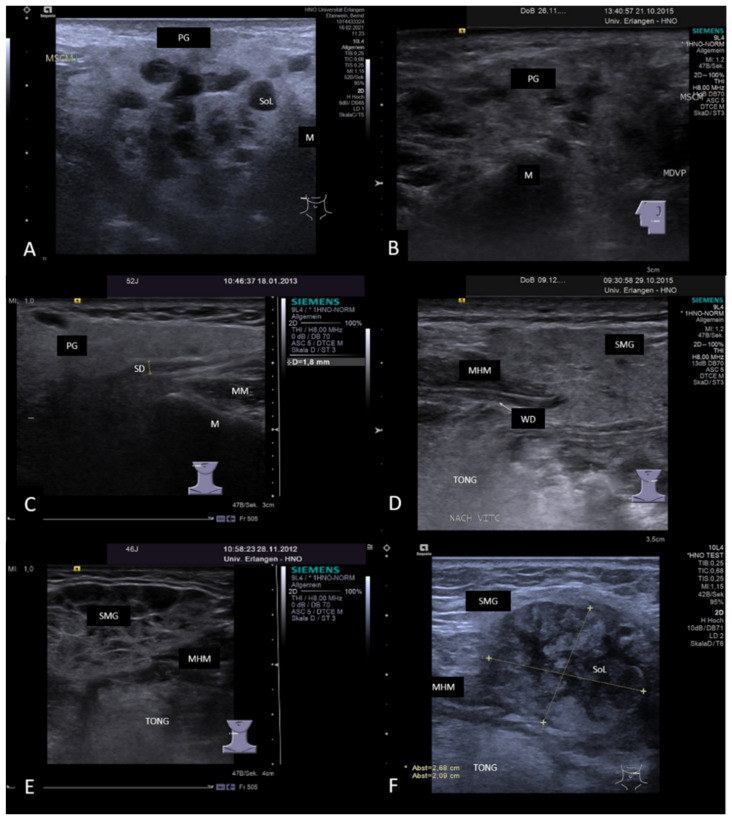
(**A**–**E**): Ultrasound images in histology-proven IgG-4-related salivary gland disease showing the variability of the findings in PGs and SMGs. In Figures (**A**,**B**), the parenchyma of PGs of the left side shows multiple hypoechoic to anechoic space-occupying lesions, while the residual gland parenchyma itself was not significantly changed (**A**) or hypoechoic (**B**). These presumably mainly represent an infiltration of lymphatic cells. The gland parenchyma of the PG in Figure (**C**) has a fine granulated tissue texture with a slightly dilated duct (measuring 1.8 mm, **C**) similar to (**D**), where a SMG on the left side is depicted. In this gland, the diffuse changes appear more pronounced. In both cases, the glands are not significantly enlarged. (**E**) shows an enlarged SMG on the right side. The parenchyma is characterized by hypoechoic honeycomb-like changes due to the infiltration of the cells of the lymphatic system. As Figure (**F**) illustrates, tissue changes may also look like a tumor: a heterogeneous, irregular shaped, but sharply outlined space-occupying lesion measuring 2.68 × 2.09 cm is visible. Histologic finding after submandibulectomy revealed an IgG4-associated xanthogranulomatous sialadenitis (>150 positive cells per 1 HPF) with prominent histiocytic reaction. Note that the gland tissue surrounding the lesion does not appear pathologically changed. Abbreviations: PG, parotid gland; SMG, submandibular gland; M, mandible; MM, masseter muscle; MHM, mylohyoid muscle; MDVP, digastric muscle venter posterior; MSCM, sternocleidomastoid muscle; TONG, tongue; SD, Stensen’s duct; WD, Wharton’s duct; SoL, space occupying lesion.

IgG4-associated sialadenitis is an autoimmune disease characterized by a massive infiltration of IgG4-producing plasma cells into different organs including the major and minor salivary glands. It more often affects SMGs than PGs, more often bilateral than unilateral glands, and in some cases, all major salivary glands are involved. More than 30–40% of the patients are diagnosed as having an IgG4-related disease present with sialadenitis [167,168,169]. The value of ultrasound was investigated in reports published in recent years with characteristic findings described [76,163,169,170,171,172,173]. IgG4-associated sialadenitis may be considered as a “chameleon” concerning its variability of US findings. In one report, five patterns of ultrasound features were described: superficial hypoechoic, multiple hypoechoic foci, whole-gland heterogeneity, space occupying, and normal echo [171]. Our experiences in IgG4-associated sialadenitis confirm this variability in US. The tissue texture of the parenchyma can appear hypoechoic only to a slight or diffuse and pronounced extent. It may also sometimes have a coarse aspect, a reticular or even a nodular tissue- or tumor-like pattern. Ducts can be dilated to a lesser or more pronounced extent, but megaducts are also observable (Figure 16A–E).

**Figure 17:**
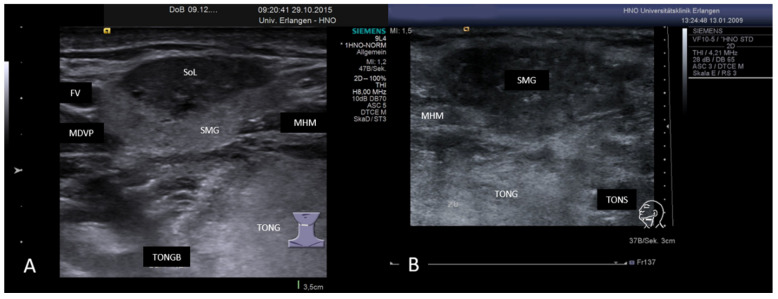
(**A**,**B**) Sclerosing IgG-4-related sialadenitis in two SMGs. In (**A**), the caudal part of the right-sided SMG is characterized by a mixed hypoechoic to anechoic and hyperechoic tissue pattern. In (**B**), the whole gland is changed in this way. A functioning parenchyma is no longer visible. Clinical diagnosis was first presence of a “Küttner’s tumor”. Abbreviations: SMG, submandibular gland; MHM, mylohyoid muscle; MDVP, digastric muscle venter posterior; TONG, tongue; TONGB, base of tongue; TONS, tonsil; FV, facial vein; SoL, space occupying lesion.

As clinical symptoms are often unspecific, a distinct proportion of the patients present with a hard nodule in the submandibular space. In these cases, ultrasound shows changes in the submandibular gland characteristic for chronic fibrotic sialadenitis. The gland is hard on palpation and smaller. The parenchyma appears prominently hypoechoic, but in some cases can also be heterogeneous with hyperechoic reflexes. This constellation of findings can represent the final state of unspecific chronic sialadenitis caused by IgG4-related disease. A substantial part of similar changes observed in the SMG, previously termed as “Kuettner’s tumor” [113], may in fact be chronic IgG4 disease-related sialadenitis. These pronounced hypoechoic changes may also involve only parts of the gland (Figure 17A,B), and then focal chronic sialadenitis must be differentiated. In the latter case, however, the regular gland anatomy and perfusion is unchanged (see above, Figure 2).

Elastography showed that stiff elastographic changes of hypoechoic areas within the parenchyma, which indicate increased stiffness of the tissue, presumably caused by IgG4-positive lymphocytes, rapidly improved after systemic treatment with cortisone as a sign of recovery of the gland, indicating that monitoring after treatment seems possible [76].

If US is used in IgG4-related sialadenitis, it is a very useful tool for suspecting this disease, but the final diagnosis can only be made based on clinical and serological findings and on gland biopsy [168,173]. Due to the variable appearance in US, the DD includes the whole spectrum of subacute or chronic sialadenitis with sialodochitis. Ning et al. used two different scoring systems in patients with SS and IgG4-related disease. They reported that scores were higher in IgG4-related sialadenitis compared to SS and that semiquantitative US scoring systems can not only evaluate and quantify the lesions of salivary glands in IgG4-related sialadenitis but also be helpful in the differential diagnosis of SS [172].

#### 3.4.5. Sarcoidosis with Sialadenitis

**Figure 18:**
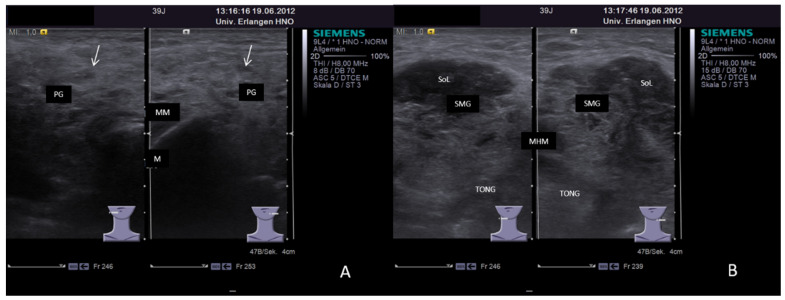
(**A**,**B**) Thirty-nine-year-old patient with involvement of all glands by histology-proven sarcoidosis. The parenchyma of the PGs has a heterogeneous, more fine-granulated and coarse aspect caused by diffuse hypoechoic areas, supposably caused by infiltration of granulocytes (white arrows, **A**). The SMGs are enlarged by diffuse hypoechoic infiltration, which was focally pronounced in the caudal part (**B**). Abbreviations: PG, parotid gland; SMG, submandibular gland; M, mandible; MM, masseter muscle; MHM, mylohyoid muscle; TONG, tongue; SoL, space occupying lesion.

Out of the specific diseases with possible involvement of salivary glands, granulomatous diseases should also be mentioned. Sarcoidosis mostly involves bilateral glands and may involve all major salivary glands, but can be only be present unilaterally. Both PGs and/or SMGs can be affected [174,175,176]. Salivary gland involvement, also including the presence of Heerfordt-syndrome, is rare. US experiences are rare, and particularly, findings in salivary glands in this disease were rarely reported [31,153]. Ultrasound shows enlargement of the gland parenchyma. Due to the invasion of granulocytes, diffuse or focal slight or pronounced hypoechoic changes are visible, and the parenchyma is heterogeneous with a more coarse than cloudy tissue texture. Vascularity is not notably affected (Figure 18A,B).

A hallmark of sarcoidosis is lymphadenopathy. Peri- and intraglandular lymph nodes are mostly increased. The diagnosis can be suspected in connection with cervical and mediastinal lymphadenopathy, further clinical signs and increased calcium and ACE levels in serology. DD should include intra- or periglandular lymphadenopathies and lymphoma of the salivary gland. With respect to possible focal changes in the gland parenchyma, the eosinophilic hyperplastic lymphogranuloma (aka Kimura’s disease) should also be considered, as the latter may also cause focal or diffuse hypoechoic, heterogeneous changes in the gland parenchyma. If subcutaneous nodules, lymphadenopathy, and increased IgE-levels are present, Kimura’s disease should be excluded [177,178]. In focal changes of sarcoidosis, focal chronic sialadenitis must also be distinguished. In contrast to the former, the tissue texture and perfusion is regular in the latter condition (see above, Figure 2).

In summary, it must be highlighted that the mentioned diseases show some specific, but also less specific changes in US. US can be used to suspect a diagnosis but does not always allow establishing an exact DD. In particular, chronic sialodochitis may be difficult to distinguish from a (low grade) duct stenosis. Focal infiltration in IgG4-related disease and sarcoidosis is another critical point, and both must be differentiated from lymphoma. This has to be finally made by additional blood tests, histopathological examinations, and imaging extending beyond the head and neck region. The possibilities and the limits of US in the described diseases/conditions are summarized in Table 2. If the possibilities are considered, US can be regarded as an excellent method for primary screening, for making a suspicion diagnosis, for initiation of further management, and for monitoring in the follow-up of these conditions.

### 3.5. Ultrasound in Monitoring Gland Function at the Follow-up Examination in Inflammatory and Obstructive Salivary Gland Disease

**Figure 19:**
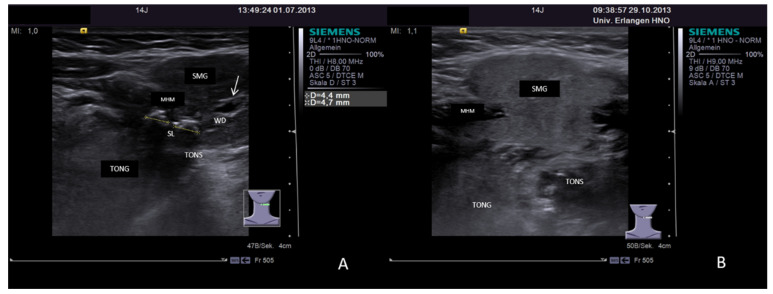
(**A**,**B**) Case of sialolithiasis of left-sided SMG in a 14-year-old patient demonstrating post-treatment recovery of the gland. Pre-treatment status shows two sialoliths within the hilum of the gland measuring 4.4 and 4.7 mm. The gland parenchyma is enlarged due to congestion with central duct dilation (white arrow, **A**). Post-treatment US nearly 4 months later demonstrates recovery of the gland. The parenchyma turned out to a have a nearly normal, slightly hyperechoic tissue texture, and no duct obstruction, and congestion is no longer visible (**B**). Abbreviations: SMG, submandibular gland; MHM, mylohyoid muscle; TONG, tongue; TONS, tonsil; SL, sialolithiasis.

Post-therapeutic recovery of the gland after the treatment of inflammatory and/or obstructive diseases of the major salivary glands can be observed in 85–90% of cases [179,180,181]. According to our experiences after the treatment of such diseases, recovery of the gland function can be observed and monitored by US. Hallmarks of a recovery of the gland function are a change in the tissue texture of the parenchyma from hypoechoic to slightly hyperechoic (Figure 19A,B).

In addition to this, as mentioned earlier, the state of the secretory function of the gland can be roughly estimated after stimulation of the gland secretion [106,107]. If duct dilation can be observed directly after stimulation, it can be assumed that secretory function is present and not seriously compromised. Elastography may also be an interesting tool for monitoring the recovery of the gland parenchyma after the treatment of chronic obstructive sialadenitis [74,77].

**Figure 20:**
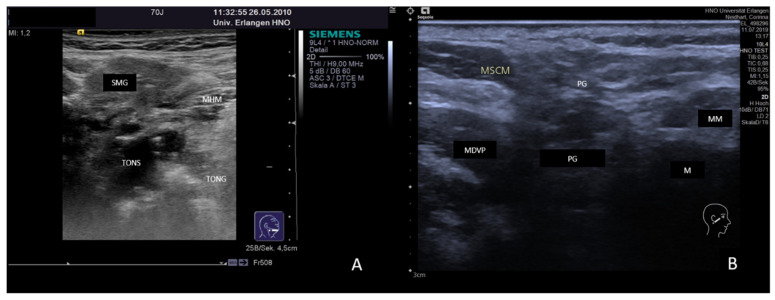
(**A**,**B**) Atrophy of the SMG after sialolithiasis (**A**) and of the PG after postoperative radiotherapy of the head and neck region (**B**). The gland parenchyma shows a markedly heterogeneous aspect. The SMG hyperechoic reflexes are presumably caused by scarring tissue, and hypoechoic areas indicating chronic inflammation are recognizable, which led to shrinkage of the gland volume (**A**). The image of the right-sided PG shows nearly no typical gland tissue any more. A degeneration of the parenchyma is visible, which is replaced by hypoechoic areas (presumably corresponding to fatty tissue) and by hyperechoic areas (presumably corresponding to fibrous tissue). Abbreviations: PG, parotid gland; SMG, submandibular gland; M, mandible; MM, masseter muscle; MSCM, sternocleidomastoid muscle; MHM, mylohyoid muscle; MDVP, digastric muscle venter posterior; TONG, tongue; TONS, tonsil.

Gland atrophy, caused by longstanding inflammatory or obstructive disease (Figure 20A), by irradiation of the head and neck region (Figure 20B) or by radio-iodine treatment due to carcinoma of the thyroid gland (see above), can be reliably diagnosed by US. Gland atrophy occurs mostly unilaterally, the gland volume is reduced, and the parenchyma has a mixed echogenicity due to fibrous (hyperechoic) and fatty (hypoechoic) degeneration (Figure 20A,B). Saliva secretion is not noted or cannot be provoked, and patients do not report relevant complaints.

Elastography demonstrated an increase in shear wave velocity within SMGs after radiation of the head and neck region and may be used as an indicator for structural changes indicating gland atrophy [75].

## 4. Conclusions

US is a valuable diagnostic tool in obstructive salivary gland diseases such as sialolithiasis and stenosis. The variable diseases associated with sialodochitis cannot always be distinguished with US. Primary inflammatory diseases like crjP, SS, IgG4-related disease or sarcoidosis show some specific features but can also share some sonographic signs. In these cases, the final diagnosis can be made only after the inclusion of an anamnesis, clinical symptoms, and serologic blood tests and by histopathologic investigation after gland biopsy (Table 2). Nevertheless, in the hands of the treating physician, US may not only be an excellent screening method, but also be used for adequately diagnosing a suspicion and improving further management.

## Figures and Tables

**Table 1:** Ultrasound Features of Stenoses Associated with Ductal Anomalies and Stenoses without Duct Anomalies (according to Goncalves and Koch et al. [147]).

Stenoses	Ultrasound Feature				Duct Dilation (mm)
	Webs	Duct Kinking	Megaducts	Normal Echogenicity	
Without duct anomalies (*n* = 90)	7.8% (*n* = 7)	3.3% (*n* = 3)	15.6% (*n* = 14)	25.6% (*n* = 23)	3.27 ± 2.17 (*n* = 90)
With duct anomalies (*n* = 40)	82.5% (*n* = 33)	75.0% (*n* = 30)	87.5% (*n* = 35)	77.5% (*n* = 31)	9.19 ± 3.65 (*n* = 40)
Fisher’s exact test/χ^2^ test	*p* < 0.001	*p* < 0.001	*p* < 0.001	*p* < 0.001	*p* < 0.001

**Table 2:** Important US features in non-sialolithiasis caused infectious, inflammatory, obstructive diseases in salivary glands. Limits of US diagnosis are indicated by additional diagnostics needed.

Disease	VD	HIV	craS (SD) °	craS (Sten) °	ESD	cjrP	SS	IgG4	SA	RTiS/RITiS
I. US-Feature										
Gland volume	↑	↑	↑ ^#^	↑ optional ↓ (late stages) ^#^	↑	↑	↑ optional ↓ (late stages)	↑ optional ↓ (late stages)	↑	↑ (early stages) ↓ (late stages)
Parenchyma: echopoor	optional	no	yes	yes	yes	yes	yes	yes (focal)	no	yes
Parenchyma: heterogeneous	no	no	no	optional	optional	yes	yes	yes	yes	yes (late stages)
Tissue aspect: spongy	yes	no	no	no	no	no	no	no	no	no
Tissue aspect: leopard-skin-like	no	no	no	no	no	yes	yes	optional	no	no
Tissue aspect: polycystic	optional	yes	optional	optional	yes	yes	optional (late stages)	no	no	no
Tissue aspect: echopoor foci	no	no	no	no	no	no	yes	no	no	no
Tissue aspect: coarse	no	no	no	no	no	no	no	optional	yes	no
Tissue aspect: honey-comb-like	no	no	no	no	no	no	yes (acute progress)	optional (focal infiltration)	optional (focal infiltration)	no
Tissue aspect: space occupying lesion-like	no	no	no	no	no	no	optional (MALT-lymphoma)	optional (focal infiltration)	optional (focal infiltration)	no
Tissue aspect: atrophy	no	no	no ^#^	optional (late stages) ^#^	no	optional (late stages)	yes (late stages)	yes (late stages)	no	yes (late stages)
Tissue aspect: Perfusion increased	yes	no	optional	optional	optional	optional	optional	optional	no	no
Duct dilation	no	no	yes	yes	yes	no	no	optional	no	optional
Formation of megaduct	no	no	optional	optional (no duct anomaly); yes (with duct anomaly)	yes	no	no	optional	no	no
Lympadenopathy: significant	optional	optional	no	no	no	no	Optional (MALT)	no	yes	no
II. Additional diagnostics: Blood tests	no	Yes (PCR)	no	no	yes (eosinophils)	no	yes (ANA, SS-A, SS-B)	yes (IgG4)	yes (ACE, Calcium)	no
III. Additional diagnostics: Gland biopsy	no	no	no	no	yes (eosinophilic inflammation)	no	yes (ANA, SS-A, SS-B)	yes (IgG4:IgG > 40%)	yes	no
IV. Additional diagnostics: LN-biopsy	no	no	no	no	no	no	no *	no	yes	no
V. Additional diagnostics: Imaging (CT, MRI)	no	no	no	no	no	no	no	CT human/MRI human	CT Thorax	no

Abbreviations: VD, Viral disease; craS (SD), unspecific chronic recurrent sialadenitis with sialodochitis; craS (Sten), unspecific chronic recurrent sialadenitis with stenosis; ESD, eosinophilic SD (Kußmaul´s disease); cjrP, chronic juvenile recurrent parotitis; HIV, Human immune-deficiency virus infection; SS, Sjoegren´s syndrome; IgG4, IgG4-asscoiated disease; SA, Sacroidosis; RTiS/RITiS, radiotherapy/radioiodinetherapy-induced sialadenitis; Legends: ° representative for bacterial infection as congestion is always caused accompanied by bacterial infection; ^#^ transition from SD to Sten possible; * not for primary purposes, but for diagnostics of MALT-lymphoma.

## Data Availability

Not applicable.
